# Impact of Extraction Parameters on the Recovery of Lipolytic Activity from Fermented Babassu Cake

**DOI:** 10.1371/journal.pone.0103176

**Published:** 2014-08-04

**Authors:** Jaqueline N. Silva, Mateus G. Godoy, Melissa L. E. Gutarra, Denise M. G. Freire

**Affiliations:** 1 Departamento de Engenharia Bioquímica, Escola de Química, Universidade Federal do Rio de Janeiro, Rio de Janeiro, Brazil; 2 Departamento de Bioquímica, Instituto de Química, Universidade Federal do Rio de Janeiro, Rio de Janeiro, Brazil; 3 Departamento de Engenharia Bioquímica, Escola de Química/Polo Xerém, Universidade Federal do Rio de Janeiro, Rio de Janeiro, Brazil; University of Nottingham, United Kingdom

## Abstract

Enzyme extraction from solid matrix is as important step in solid-state fermentation to obtain soluble enzymes for further immobilization and application in biocatalysis. A method for the recovery of a pool of lipases from *Penicillium simplicissimum* produced by solid-state fermentation was developed. For lipase recovery different extraction solution was used and phosphate buffer containing Tween 80 and NaCl showed the best results, yielding lipase activity of 85.7 U/g and 65.7 U/g, respectively. The parameters with great impacts on enzyme extraction detected by the Plackett-Burman analysis were studied by Central Composite Rotatable experimental designs where a quadratic model was built showing maximum predicted lipase activity (160 U/g) at 25°C, Tween 80 0.5% (w/v), pH 8.0 and extraction solution 7 mL/g, maintaining constant buffer molarity of 0.1 M and 200 rpm. After the optimization process a 2.5 fold increase in lipase activity in the crude extract was obtained, comparing the intial value (64 U/g) with the experimental design (160 U/g), thus improving the overall productivity of the process.

## Introduction

The market for lipases is growing steadily, mostly because of their capacity to catalyze hydrolytic and synthesis reactions using a large range of substrates, and also because they sometimes demonstrate regioselectivity and enantioselectivity [13]. As such, lipases have been widely used in several fields, including the detergent, pharmaceutical and fine chemical industries [23].

Enzymes of industrial interest have traditionally been produced by submerged fermentation (SmF). However, solid-state fermentation (SSF) is being used for the production of enzymes due to its advantages over SmF, such as its high productivity, generation of high-quality products, and use of agro-industrial waste, an abundant and low-cost raw material [26]. The use of agro-industrial waste/complex matrix as a culture medium also induces the production of different hydrolytic enzymes such as amylases and proteases in the same fermentation batch [26], or even pools of lipases with different catalytic properties [6].

In the SSF of agro-industrial waste such as babassu cake, the fungi grow on the surface or inside the solid particles, breaking down their complex nutrients by secreting enzymes and consuming their monomers [11]. In SSF, extracellular enzymes remain absorbed in the fermented solids as the liquid phase within the particles, and mass transfer in SSF is reduced [19]. Lipases produced by SSF can be employed in the liquid form or as fermented solids for the production of biodiesel [22], as described by Salum (2010). However, for almost all industrial applications, lipases are used in liquid solutions and can be concentrated, purified and immobilized in polymeric supports before its use [16]. Then, in SSF the step of enzyme extraction is as important as the step of enzyme production, requiring attention. The production of lipases by SSF has been studied by many authors using different culture media, such as babassu cake [11], wheat bran [17], soybean meal [20] and castor bean waste [8], but only a few studies have been done into the extraction of enzymes from fermented solids, and all of these show that the optimization of this step significantly enhances the overall productivity of the process. The type of extraction solution, temperature, stirring rate, solid/liquid ratio and pH are the main parameters studied for the extraction of xylanase [12], pectinases [3] and proteases [1]. The extraction of lipases produced by the solid-state fermentation of soybean meal was studied and temperature was found to be the most important factor [26].

The fungus *Penicillium simplicissimum* produces high hydrolytic lipase activity in SSF systems using babassu cake, and the lipases present in the crude extractant have attractive catalytic properties such as thermostability and thermophilic characteristics [9]; [11]. Its crude extract has been successfully applied to the biological/enzymatic treatment of liquid fish waste [25]. Moreover, lipases from this fungal strain produced by the SSF of babassu cake show enantioselectivity when immobilized on supports with different degrees of hydrophobicity, indicating the presence of at least three lipases [4].

The aim of this work was to select suitable solutions for the extraction of a pool of lipases from *P. simplicissimum* produced by the solid-state fermentation of babassu cake; the identification of parameters that statistically influence the process of lipase extraction, using Plackett & Burman experimental design (PB); and process optimization using Central Composite Rotatable Design (CCRD), identifying the extract conditions that yield the highest lipase activity. The optimization of the extraction step could enhance productivity and thus reduce the cost of lipase production by this fungus.

## Materials and Methods

### Microorganism

*Penicillium simplicissimum* was selected for use as it is a good lipase producer by SSF [9]. To obtain the spores, the fungus was propagated in an incubator for 7 days at 30°C, in a medium containing (% w/v): 0.5 (NH_4_)_2_SO_4_; 2.0 soluble starch; 0.025 MgSO_4_·7H_2_O; 0.05 KH_2_PO_4_; 0.5 CaCO_3_; 0.1 yeast extract; 1.0 olive oil; and 2.0 agar. The spores were suspended in phosphate buffer (50 mM, pH 7.0). The spore suspension was further used during the SSF. The spore concentration was determined by counting using an optical microscope and a Neubauer chamber.

### Solid-state fermentation

Solid waste from the extraction of babassu oil, called babassu cake (TOBASA S.A), was used as the culture medium. The solids were supplemented with 6.25% (w/w) molasses (Usina Santa Elisa, Campinas, SP) and 65% moisture in wet basis(w/w). The cake was ground and sieved to obtain different particle sizes (up to 3.0 mm). Tray-type reactors (Polipropylene, diameter 10 cm, not perforated) were used with 15 g dry solids, which were inoculated with 10^7^ spores/gram of dry solid and incubated in a chamber with temperature and relative humidity set to 30°C and 95%, respectively, for 72 h [9].

### Enzyme recovery

#### Determination of extraction time of the pool of lipases

The enzyme was recovered using standard conditions (5 mL/g sodium phosphate buffer (0.1 M, pH 7.0), agitation at 200 rpm, 35°C) for 0, 10, 20, 30, 40 and 60 minutes. Next, the cake was pressed to obtain the crude enzyme extract, which was centrifuged at 3000x*g* for 5 minutes. The supernatant was used to determine lipase activity. The supernatants obtained from 10, 20, 30 40 and 60 min. extraction time were lyophilized and resuspended in a sample buffer, without SDS and b-mercaptoethanol, at the same protein concentration, then submitted to non-denaturing PAGE and subsequently zymography [15].

#### Selection of extraction solution

5 mL sodium phosphate buffer (0.1 M, pH 7.0) was added per gram of babassu cake, followed by shaking at 200 rpm and 35°C (standard condition) for 40 minutes to guarantee total lipase extraction. Sodium phosphate buffer with NaCl (0.6% (w/v)), Tween 80 (0.1% (w/v)), Triton X-100 (0.5% (w/v)) and glycerol (20% (w/v)) was also used as an extraction solution. Lipase activity was determined using the supernatant and then the lipase was stored at −20°C.

#### Determination of lipase recovery conditions

The most important factors for lipase extraction were screened using Plackett & Burman (PB) experimental design. The factors studied were: stirring rate (100–300 rpm), volume of extraction solution (5–9 mL/g), temperature (25–45°C), buffer molarity (0.01–0.1 M), NaCl concentration (0–0.2%w/v) and Tween 80 concentration (0–0.2%w/v).

Subsequently a Central Composite Rotatable Design (CCRD) was used to optimize the extraction conditions as a function of the important factors identified in the PB design. The factors studied were: volume of extraction solution (3–7 mL/g), temperature (25–35°C), pH (6.0–8.0) and Tween 80 concentration (0.1–0.5%w/v), keeping the buffer molarity constant at 0.1 M, the stirring rate at 200 rpm and with no addition of NaCl.

For all these experiments it took 40 minutes to guarantee that all the lipase had been extracted.

### Determination of lipase activity

Lipase activity was determined by the spectrophotometric method described by Godoy *et al* [7] with *p*-nitrophenyl laurate as a substrate. One unit of lipase activity (U) was defined as the amount of enzyme necessary to hydrolyze 1.0 µmol *p*-nitrophenyl laureate per minute under assay conditions. Lipase activity was expressed in U/g of initial dry weight.

### Zymography

The enzymes present in the crude extract with lipase/esterase activity were detected by zymography following non-denaturing PAGE [15]. After electrophoresis, the gel was treated with α-naphthyl acetate 0.02% (w/v) and Fast Blue RR salt 0.05% (w/v) in 0.1 M sodium phosphate buffer, pH 6.2, revealing bands with lipase/esterase activity [14].

## Results and Discussion

### Determination of extraction time

**Figure 1:**
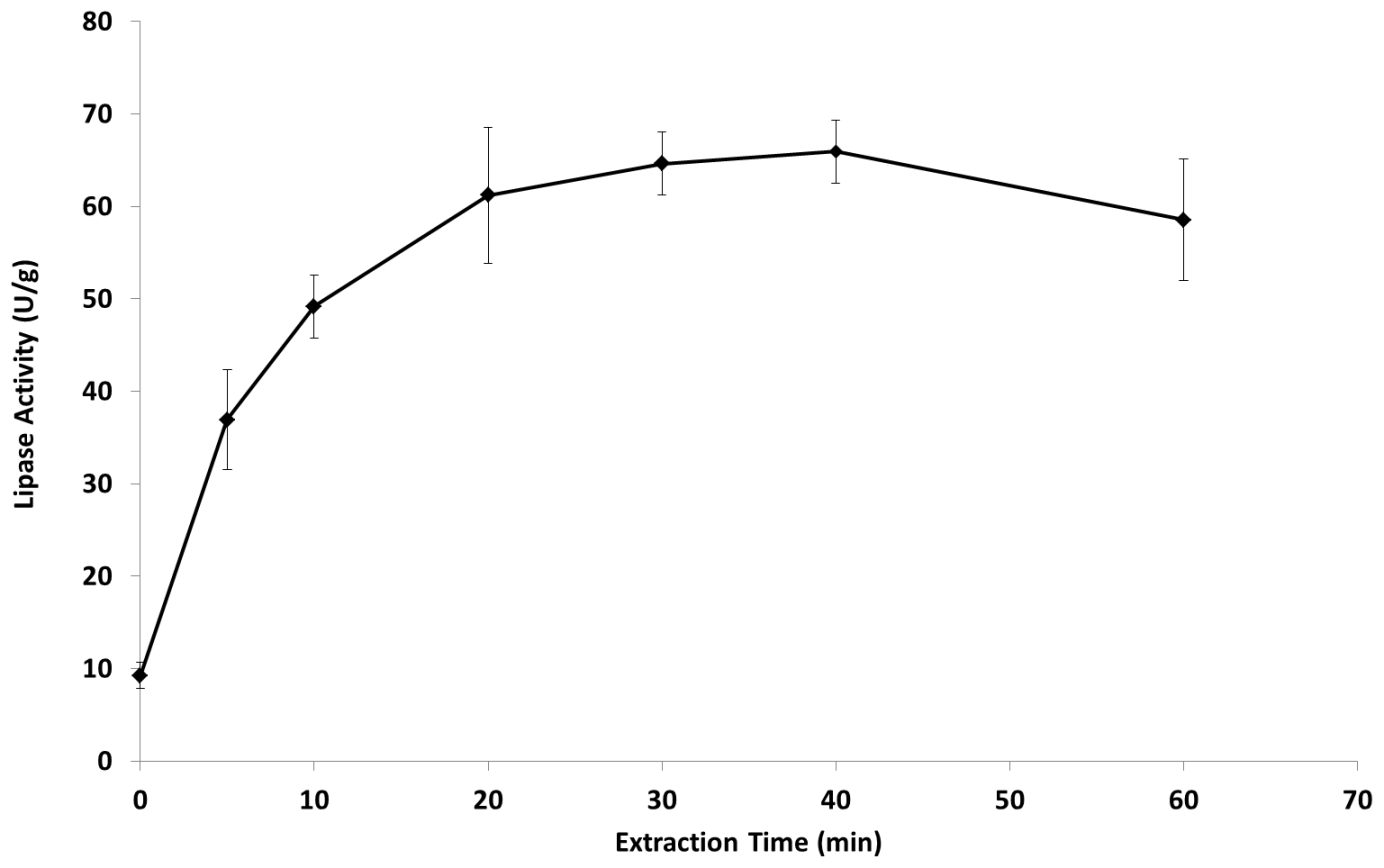
Kinetic of lipase extraction in sodium phosphate buffer (0.1 M, pH 7.0) with agitation of 200 rpm.

There was an increase in lipase extraction up to 20 minutes, after which no time difference was observed in the recovery of the enzymes, considering the standard error (Figure 1). This indicates that 20 minutes contact time is long enough for the complete extraction of lipases from *P. simplicissimum*. Aikat *et al* [1] studied the protease recovery profile from fermented wheat bran and found that 90% of protease recovery was obtained in 2 h extraction and that extraction was complete after 10 h. In this study it was possible to recover 100% of lipase in just 40 minutes. For lipases from fermented soybean meal, almost all lipase extraction occurs in the first two minutes of the extraction process, a fact the authors attribute to the growth and secretion of enzymes by the fungus at the surface of the solid particles [25]. In our case, extraction took approximately 10 times longer than reported by Vardanega *et al*. (2008) [24], because *P. simplicissimum* not only grew on the surface of the babassu cake, but also effectively penetrated the solid particles [11], making it harder to extract the lipases secreted within them. Another hypothesis is that the lipases from *P. simplicissimum* bind or interact more strongly with some components at the matrix surface and/or the fungal wall, based on the hydrophobic nature of lipases and fungal surface in SSF (MUNOZ 1995), making it take longer to extract them.

**Figure 2:**
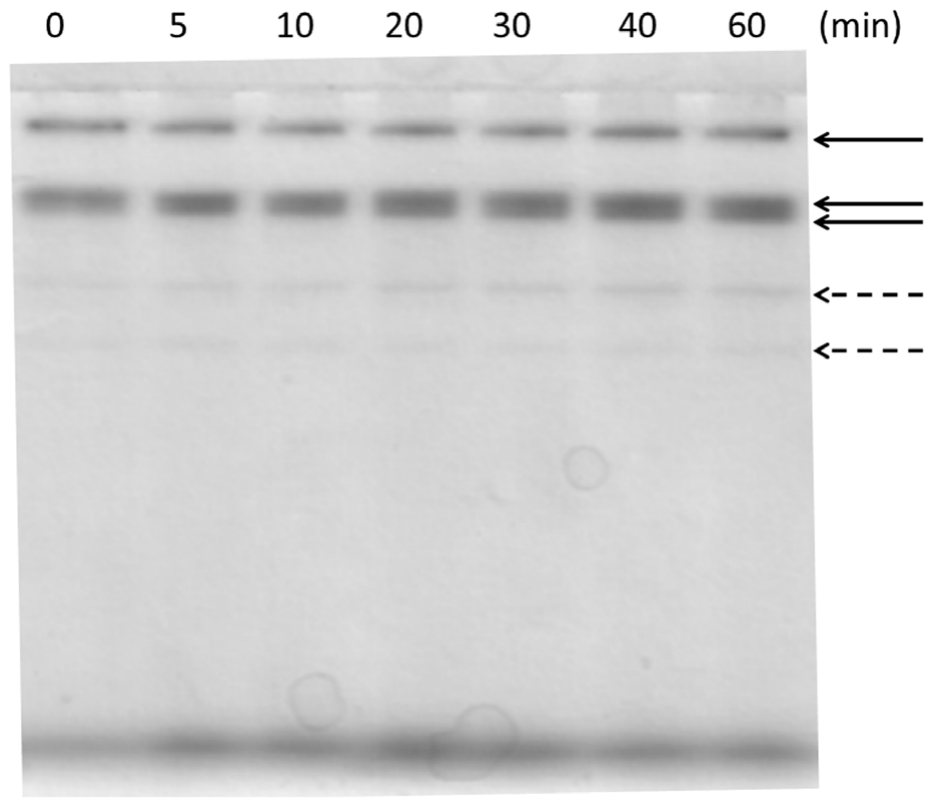
Zymography of a non-denaturing PAGE using α-naphtyl acetate as substrate showing lipase extraction profile major esterase/lipase bands (non-dashed arrows); weak esterase/lipase bands (dashed arrows).

To determine the number of different lipases produced by *P. simplicissimum* in the SSF of babassu cake and the extraction profile of each lipase, non-denaturing PAGE was performed by adding 30 µg protein per lane, followed by a zymogram using α-naphthyl acetate as a substrate. Three major bands with lipase/esterase activity were revealed (Figure 2 – non-dashed arrows). When the SSF of castor bean waste was undertaken, nine lipase/esterase bands were observed [7] with the same *Penicillium* strain, indicating that the type of matrix and fermentation can induce different enzymes or proportions thereof, producing extracts with different catalytic properties. In this work, the three major bands were extracted together for all the extraction times (Figure 2), demonstrating that they are distributed randomly on and in the matrix and probably with no differences in their interaction with the hydrophobic portion of the fungal biomass or the matrix, as they all detach at the same time. These results show that maximum lipase activity could be recovered after 20 minutes of extraction, including all the five bands with esterase/lipase activity.

### Effect of extraction solutions on recovery and enzyme storage

Four different solutions were tested, in triplicate: sodium phosphate buffer (100 mM, pH 7.0) and the same buffer with 0.6% NaCl (w/v), 0.1% Tween 80 (w/v), 0.5% Triton X-100 (w/v) and 20.0% glycerol (w/v). The samples were extracted and lipase activity was determined in fresh crude extract and after its storage at −20°C for a period of 30 days. The effect of each solution on lipase activity was tested by the addition of 0.6% NaCl (w/v), 0.1% Tween 80 (w/v), 0.5% Triton X-100 (w/v) or 20.0% glycerol (w/v) to a crude extract previously obtained by extraction with only sodium phosphate buffer and a comparison of their lipase activity were done. Our results showed that none of the solutions used interfered with lipase activity determination, guaranteeing that the effect of each solution observed later was exclusively on lipase extraction (not shown).

Tween 80, a non-ionic surfactant, proved to be an excellent enzyme recovery agent, yielding the highest extracted lipase activity (85.7 U/g). Lipases have a unique capacity to activate at the water/lipid interface due to a surface conformational change that occurs under this condition. At the water/lipid interface, the structure called the lid appears in its open form, revealing the active site. This occurs when there is adsorption of the hydrophobic pocket around the active center and the internal face of the lid onto a hydrophobic surface [18]. At the end of fermentation, the residual lipid and hydrophobin present at the fungal wall may adsorb the lipases produced [21]. As Tween 80 is a surfactant, it is possible that its hydrophobic portion attracted the lipases, promoting their desorption from the fermented solids, resulting in better extraction. Tween 80 also showed good results for the extraction of other proteins, probably because the micelles it forms surround the proteins [5]. The enzyme recovered with Tween 80 remained stable for 15 days when stored at −20°C in the crude extract form, and had an activity loss of nearly 30% after 30 days (Table 1).

**Table 1:** Activity of crude extract after extraction with different solvents and Residual activity (%) after storage of crude extract contain lipases at the temperature of −20°C.

Solvent	Activity of crude extract* (U/g)	Residual activity (%)	
		15 days	30 days
**Phosphate buffer (100 mM. pH 7.0)**	64.7±0.7	105.7	69.0
**Buffer + Tween 80 1%**	85.7±1.2	101	69.2
**Buffer + Triton X-100 0.5%**	48±1.6	101.7	64.7
**Buffer + Glycerol 20%**	63.4±0.4	91.9	65.6
**Buffer + NaCl 0.6%**	50.7±6.0	141.4	100.1

The values of lipase activity achieved afterwards the extraction were considered as 100%.

*The analyses were done in triplicate.

The stability of the lipase extracted and stored at −20°C with sodium phosphate buffer, Triton X-100 and glycerol were similar to the stability observed for Tween 80. However, when the lipase was extracted with NaCl, stability increased by about 41% (comparing with the initial activity of crude extract −50.7 U/g) over a storage period of 20 days and it was not observed any loss of activity after 30 days (Table 1).

Triton X-100 is also a surfactant, but it presented the lowest value for extracted enzyme activity (Table 1). As Triton X-100 is very viscous (240 cP at 25°C), which interferes with mass transfer, it has to be used at higher temperatures. The use of an assay temperature of 35°C could explain the low recovery efficiency.

Despite the protective effect provided by glycerol during the storage of the proteins [25], it was not found to stabilize the *P. simplicissimum* lipase at the used concentrations during the storage period, presenting the lowest residual activity after 30 days of storage (Table 1). Like Triton X-100, the high viscosity of glycerol could explain its low efficiency during lipase recovery, as shown by Castilho *et al* [2] for pectinase extraction.

The Tween 80 and NaCl solutions were selected due to their highest efficacy in the extraction and stability of *P. simplicissimum* lipases, respectively.

### Optimization of lipase extraction using experimental design

**Figure 3:**
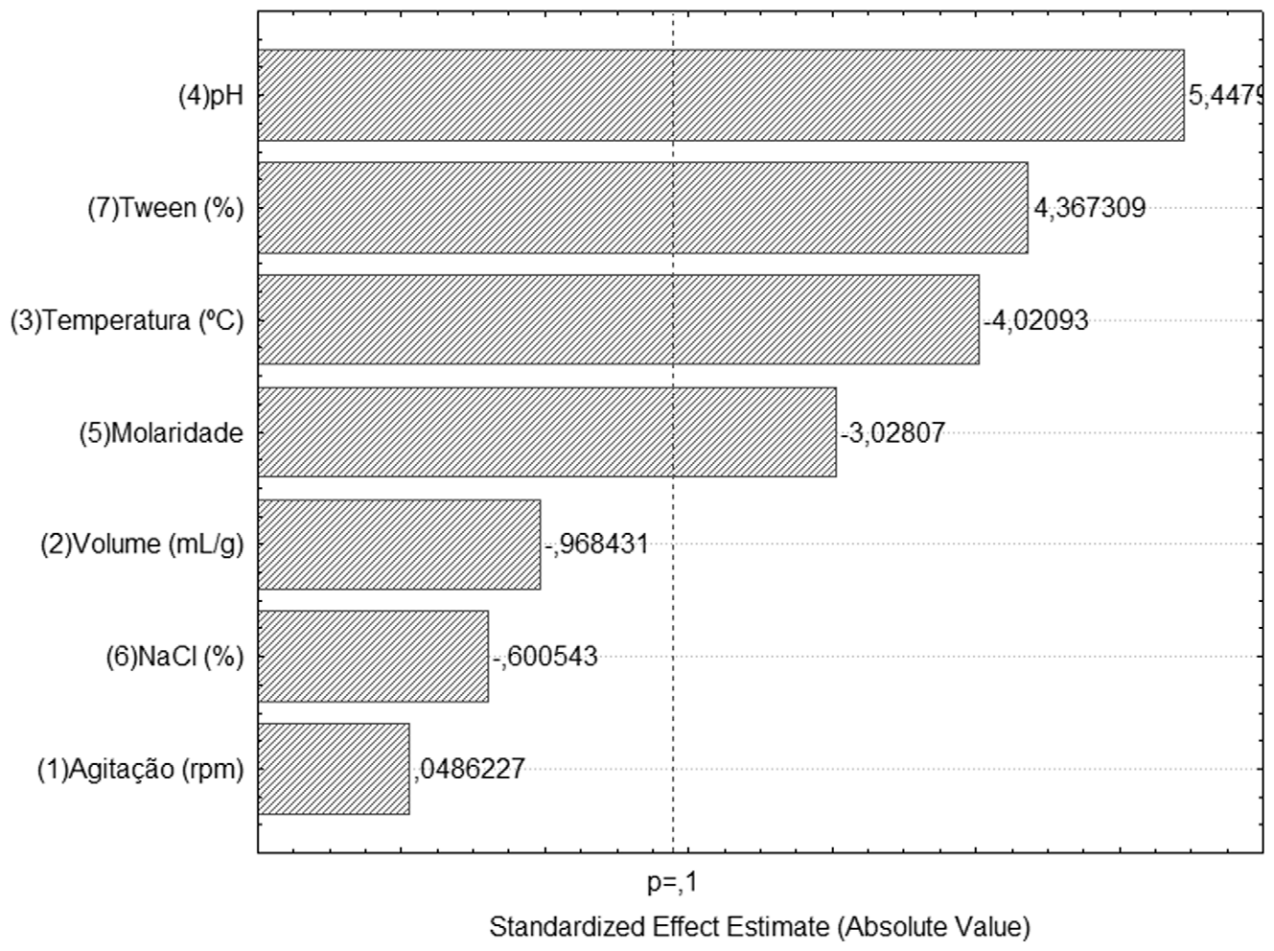
Standardized effects of studied variables and interaction on the lipase extraction, where *p* represents the significance level of the test. Statistically significant terms: *p*-value >0.1 (90% of confidence).

In the first stage of the experimental design the following variables were studied: agitation (100 to 300 rpm), volume of solvent (5 to 9 mL solvent/g dry weight of cake), temperature (25 to 45°C), pH (5.0 to 7.0), molarity of solvent (0.01 to 0.1) and concentration of NaCl and Tween 80 (0 to 0.2% (w/v)), to determine the statistically significant variables for the process. The assay conditions and values for extracted lipase activity are shown in Table 2. A Pareto chart (Figure 3) was created, which shows the standardized effects and permits the identification of the statistically significant variables for the process, with a significance level (*p*-value) of 0.1.

**Table 2:** Values for lipase activity achieved after the extraction process for the different experimental conditions (real variable values and coded levels in parenthesis) of the Plackett & Burman design.

Assays	Agitation (rpm)	Volume (mL/g)	Temp. (°C)	pH	Molarity	NaCl conc.(%)	Tween conc.(%)	Lipase activity (U/g)
1	100 (1)	5 (−1)	45 (1)	5 (−1)	0.01 (−1)	0 (−1)	0.2 (1)	58.7
2	100 (1)	9 (1)	25 (−1)	7 (1)	0.01 (−1)	0 (−1)	0 (−1)	85.9
3	300 (−1)	9 (1)	45 (1)	5 (−1)	0.1 (1)	0 (−1)	0 (−1)	11.1
4	100 (1)	5 (−1)	45 (1)	7 (1)	0.01 (−1)	0.2 (1)	0 (−1)	66.0
5	100 (1)	9 (1)	25 (−1)	7 (1)	0.1 (1)	0 (−1)	0.2 (1)	99.3
6	100 (1)	9 (1)	45 (1)	5 (−1)	0.1 (1)	0.2 (1)	0 (−1)	7.3
7	300 (−1)	9 (1)	45 (1)	7 (1)	0.01 (−1)	0.2 (1)	0.2 (1)	93.1
8	300 (−1)	5 (−1)	45 (1)	7 (1)	0.1 (1)	0 (−1)	0.2 (1)	74.9
9	300 (−1)	5 (−1)	25 (−1)	7 (1)	0.1 (1)	0.2 (1)	0 (−1)	67.5
10	100 (1)	5 (−1)	25 (−1)	5 (−1)	0.1 (1)	0.2 (1)	0.2 (1)	69.5
11	300 (−1)	9 (1)	25 (−1)	5 (−1)	0.01 (−1)	0.2 (1)	0.2 (1)	71.2
12	300 (−1)	5 (−1)	25 (−1)	5 (−1)	0.01 (−1)	0 (−1)	0 (−1)	67.0
13	200 (0)	7 (0)	35 (0)	6 (0)	0.055 (0)	0.1 (0)	0.1 (0)	84.8
14	200 (0)	7 (0)	35 (0)	6 (0)	0.055 (0)	0.1 (0)	0.1 (0)	81.0

The pH and Tween 80 concentration variables had a positive effect, indicating that a higher extraction of lipase could be obtained at the highest values used (pH 7.0 and 0.2% of Tween 80) or even higher values than those studied. On the other hand, temperature presented a negative effect, indicating that lower values should be used to improve the extraction of the enzyme. At higher temperatures, some enzymes could be denaturated, but the lipase from *P. simplicissimum* shows high stability at 50°C [10]. Another hypothesis is that high temperatures enhance the extraction of other compounds from fermented solids, which could hamper the extraction of the lipase, as reported by Diaz *et al*. [5] for the extraction of hydrolytic enzymes from fermented grape pulp.

Molarity demonstrated a significant negative effect, probably because a higher ionic strength helps the adsorption of proteins with hydrophobic surfaces, making it harder to extract them [18]. Even so, buffer molarity was set at 0.1 M, as a lower molarity value reduced the effectiveness of the buffer, affecting the pH of the final crude extract and thereby the stability of the enzyme [10]. Volume was found to have no statistical effect on lipase extraction in the tested range, and could be fixed at any value from 5 to 9 mL per gram. However, the volume/mass ratio is directly related to the product concentration, making the study of this factor in lower ranges necessary.

The second part of the experimental design consisted of optimizing the process using a Central Composite Rotatable Design (CCRD), enabling the analysis of the interaction between the variables, according to Table 3. The effects of temperature, pH, Tween 80 concentration and volume were evaluated on lipase recovery and stability when stored at -20°C for 45 days.

**Table 3:** Values of lipase activity achieved after the extraction process and residual activity after 45 days of storage at −20°C for different experimental conditions (real variable values and coded levels in parenthesis) of Central Composite Rotatable Design.

Assays	Temperature (°C)	pH	Tween (% w/v)	Volume (mL)	Lipase activity (U/g)	Residual activity (%) 45 days
1	27.5 (−1)	6.5 (−1)	0.2 (−1)	4 (−1)	68.11	97.4
2	27.5 (−1)	6.5 (−1)	0.2 (−1)	6 (1)	82.58	81.2
3	27.5 (−1)	6.5 (−1)	0.4 (1)	4 (−1)	82.78	75.3
4	27.5 (−1)	6.5 (−1)	0.4 (1)	6 (1)	86.17	81.2
5	27.5 (−1)	7.5 (1)	0.2 (−1)	4 (−1)	92.93	80.6
6	27.5 (−1)	7.5 (1)	0.2 (−1)	6 (1)	109.81	75.8
7	27.5 (−1)	7.5 (1)	0.4 (1)	4 (−1)	99.72	74.5
8	27.5 (−1)	7.5 (1)	0.4 (1)	6 (1)	106.19	80.6
9	32.5 (1)	6.5 (−1)	0.2 (−1)	4 (−1)	69.29	115.8
10	32.5 (1)	6.5 (−1)	0.2 (−1)	6 (1)	90.23	101.7
11	32.5 (1)	6.5 (−1)	0.4 (1)	4 (−1)	99.42	77.1
12	32.5 (1)	6.5 (−1)	0.4 (1)	6 (1)	96.64	87.4
13	32.5 (1)	7.5 (1)	0.2 (−1)	4 (−1)	83.46	86.1
14	32.5 (1)	7.5 (1)	0.2 (−1)	6 (1)	96.94	91.4
15	32.5 (1)	7.5 (1)	0.4 (1)	4 (−1)	90.78	81.8
16	32.5 (1)	7.5 (1)	0.4 (1)	6 (1)	111.15	78.3
17	25 (−2)	7 (0)	0.3 (0)	5 (0)	106.31	78.1
18	35 (2)	7 (0)	0.3 (0)	5 (0)	101.97	78.9
19	30 (0)	6 (−2)	0.3 (0)	5 (0)	93.89	79.8
20	30 (0)	8 (2)	0.3 (0)	5 (0)	109.22	73.4
21	30 (0)	7 (0)	0.1 (−2)	5 (0)	101.16	57.2
22	30 (0)	7 (0)	0.5 (2)	5 (0)	133.74	48.8
23	30 (0)	7 (0)	0.3 (0)	3 (−2)	81.49	53.7
24	30 (0)	7 (0)	0.3 (0)	7 (2)	110.17	59.3
25	30 (0)	7 (0)	0.3 (0)	5 (0)	95.86	57.6
26	30 (0)	7 (0)	0.3 (0)	5 (0)	106.30	71.9
27	30 (0)	7 (0)	0.3 (0)	5 (0)	82.46	86.9

A statistical analysis was carried out to estimate the effects of the variables and the interaction between them. The effects of standardized variables (*t*-values) and the significance probability test (*p*-value) were used to assess the effects of pH, Tween 80 concentration (Tw), volume (V) and temperature (T) on lipase activity. Using a 10% level of significance (*p*<0.1), it was observed that the pH (linear term), Tween 80 concentration (linear and quadratic term), volume (linear term) and pH-temperature interaction had significant effects on lipase activity.

With these experiments, it was possible to construct (through effects table – data not shown) an empirical model for lipase recovery as a function of temperature, pH, Tween 80 concentration and volume (Equation 3.1), including only the statistically significant variables with *p*<0.1.
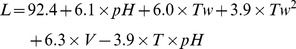
(3.1)


Where:

L  =  Lipase recovery (U/g)

pH  =  pH (codified values)

Tw  =  Concentration of Tween 80 (codified values)

T  =  Temperature (codified values)

V  =  Volume (codified values)

**Figure 4:**
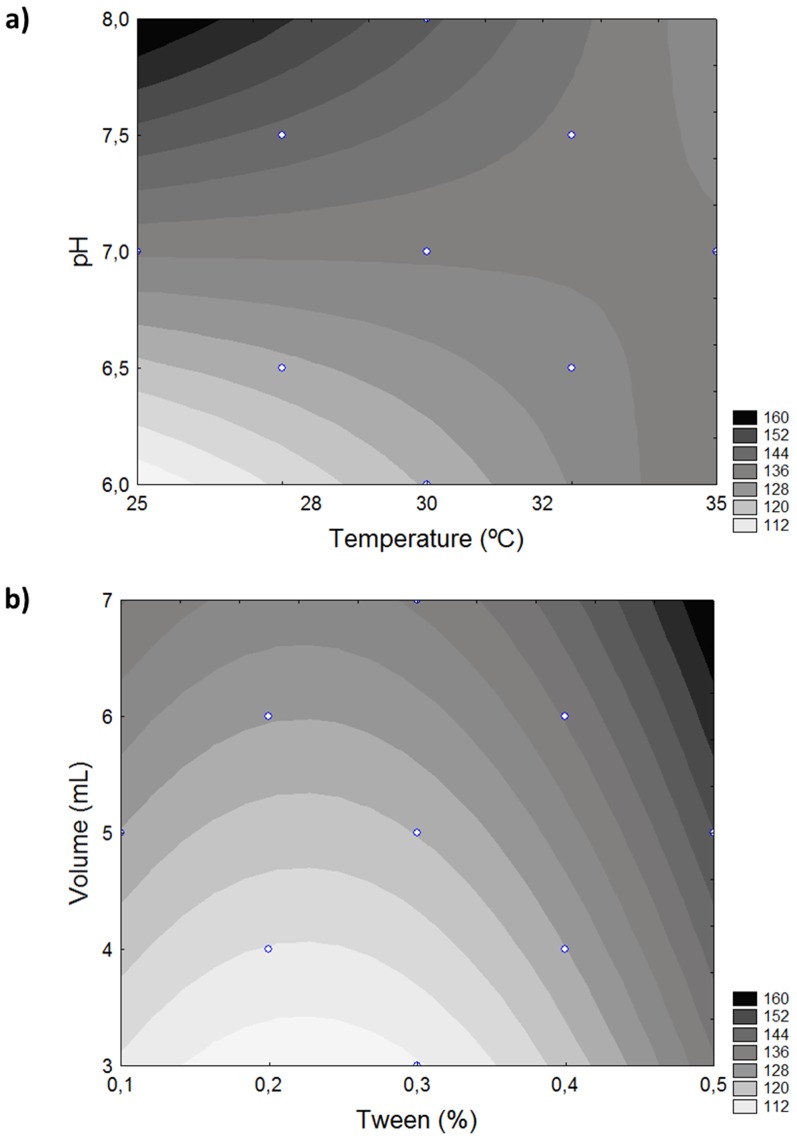
Contour plots for lipase activity in function of the variables temperature and pH (a) and volume and Tween concentration (b).

The model generated was considered predictive by analysis of variance (ANOVA), since it showed a satisfactory coefficient of determination (R^2^ = 0.7) and the F test value (7.71) was 3.6 times higher than the critical value (2.14) (Table 4). This model was used to construct contour plots, showing the values of predicted lipase activity for each condition under study (Figure 4).

**Table 4:** Analysis of variance (ANOVA) for the CCRD of enzyme extraction.

Source of variation	Sum of squares	Degrees of freedom	Means squares	F-test
Regression	3363.4	5	672.7	7.71
Residues	1830.2	21	87.2	-
Lack of fit	1544.8	19	81.3	-
Pure error	285.4	2	142.7	-
Total	5193.6	26	-	-

Regression coefficient: R^2^ = 0.65 (F_0.1;5;21_) = 2.14.

Effect and coefficient regression analysis indicated that the volume, pH and Tween 80 concentration were the most important factors influencing lipase extraction.

The results were better when the pH was higher than 7.3. Varying the pH of the extraction solution changes the protein surface charge, which probably interferes in the interaction of the enzyme with the solid matrix, making its interaction stronger or weaker. It was observed that the optimal pH for lipase extraction indicated by CCRD did not correspond to the best pH for lipase activity and stability, which were better at pH 5 and 6 [10]. As such, it is important to choose a condition which fosters high extraction without affecting enzyme stability. Similar results were observed for the extraction of lipase from soybean meal, where higher lipase extraction was obtained with pH 8.5, but the authors opted to use pH 7.0 as the enzyme was more stable under this condition [24].

Volume had a greater significance in the final enzyme concentration than in the crude extract, since one of the advantages of SSF is that it yields more concentrated products [19]. Higher extraction was attained at volumes greater than 5 mL, indicating that at lower volumes the solvent was quickly impregnated by proteins and products from the hydrolysis of this agro industrial waste, preventing the lipases from being extracted. In this case the solution is saturated with solutes that diminish mass transfer. Aikat *et al* [16] observed this effect for protease extraction, finding that 5 mL/g was the optimal condition. Vardanega et al. [24] obtained better results for the lowest volume of extraction solution tested (4 mL/g), yielding a more concentrated crude extract.

The addition of Tween 80 to the extraction solution had a positive effect, indicating that higher concentrations of this surfactant would promote greater lipase extraction. This is probably due to the hydrophobicity of lipases, enabling the lipase to be desorbed from the matrix by affinity with Tween 80, and/or the surfactant property of Tween 80, by which it surrounds the proteins extracted during micelle formation, enabling extraction. A similar positive effect was also observed by Díaz *et al*. [5] for the extraction of xylanase. The effect of Tween 80 on lipase extraction had not been previously studied.

The best results were obtained at temperatures lower than 30°C. One hypothesis is that at higher temperatures, other compounds could be extracted from the babassu cake, saturating the solution more quickly and hampering the extraction of lipases. The highest temperature tested in the experimental design (35°C) probable do not cause lipase denaturation effects because lipase from *P. simplicissimum* on in the crude extract is highly stable up to 50°C (half-life of 5 hours at pH 5.0) [10]. Vardanega *et al* [24] also achieved higher lipase extraction at a lower temperature (25°C), but in this case the *Penicillium* sp. lipase was more stable at the same temperature.

Through the analysis of the results of this experimental design, it was possible to obtain maximum values of lipase activity at temperatures from 25 to 30°C, pH from 7.3 to 8.0, volume from 5 to 7 mL/g dry weight, and Tween 80 concentration of 0.5% (w/v). At optimal conditions no changes in the three major lipase extraction bands were observed in the zymography (data not shown).

**Figure 5:**
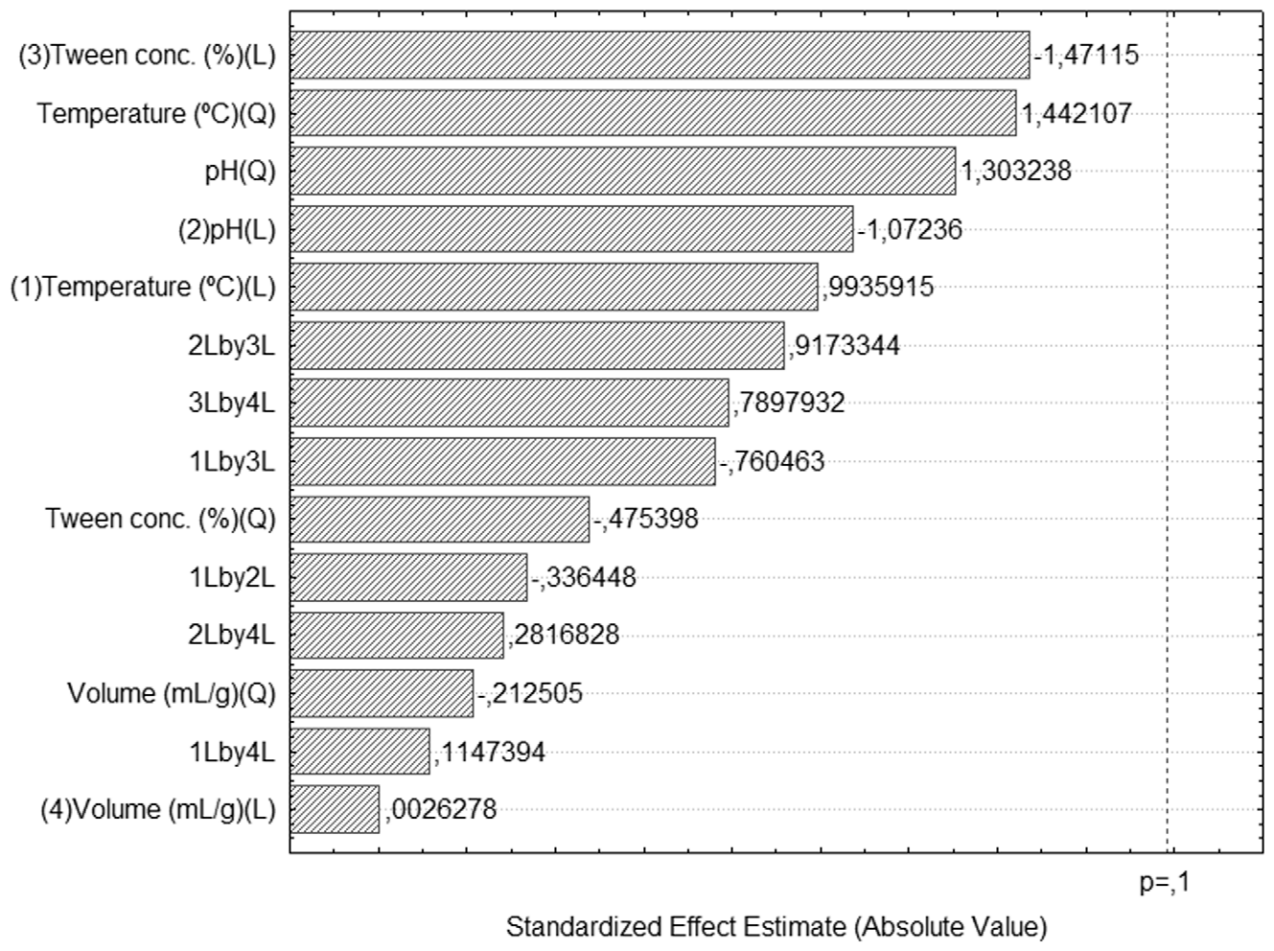
Standardized effects of studied variables and interaction on the residual lipase activity of crude extract after storage at −20°C, where *p* represents the significance level of the test. Statistically significant terms: *p*-value >0.1 (90% of confidence)

The effect of the temperature, pH, Tween 80 concentration and volume on lipase stability after the storage of the crude extract at −20°C for 45 days was also evaluated in the CCRD. The assay conditions and the residual activity (%) values after storage are shown in Table 3. The variables studied and their interactions showed no statistical effect on lipase stability (Figure 5). This indicates that the change in variable levels (in the studied range) causes no effect on lipase stability and that for any of the tested conditions approximately 78% of the residual activity can be obtained after storage at −20°C for 45 days.

The conditions for lipase extraction from soybean meal and babassu cake showed similarities at lower temperature (25°C), alkaline pH (about 8.0) and no agitation [25]. However differences in the volume/mass ratio and extraction time were observed, showing that for *P. simplicissimum* lipase extraction from babassu cake, a longer time and higher volume/mass ratio were needed.

## Conclusions

The use of 0.1% of Tween 80 in the solvent yielded higher enzyme recovery than sodium phosphate buffer (100 mM, pH 7.0), 0.6% of NaCl, 0.5% Triton X-100 and 20% glycerol. The fungus *Penicillium simplicissimum* grown in the SSF of babassu cake secretes five proteins with lipase/esterase activity, which can be extracted together from fermented solids by the proposed method. These lipases present in the crude extract proved to be stable when extracted with 0.6% NaCl and stored at −20°C for 30 days. The results after the optimization process yielded a 2.5 fold increase in the lipase activity of the crude extract (160 U/g) at 25°C, 0.5% (w/v) Tween 80, pH 8.0 and 7 mL extraction solution per gram of fermented solid, shown in the three major esterase/lipase activity bands. The use of experimental design techniques allowed a greater understanding of the effect of these variables on the extraction and stability of lipases from *P. simplicissimum*, and a significant increase in the recovery of lipases with great biotechnological potential, mainly due to this pool of enzymes high potential for hydrolysis and synthesis, and their enantioselectivity and thermostability.

## References

[1] Aikat K, Bhattacharyya BC (2000) Protease extraction in solid state fermentation of wheat bran by a local strain of Rhizopus oryzae and growth studies by the soft gel technique. Process Biochem 35: 907–914.

[2] Castilho LR, Medronho RA, Alves TLM (2000) Production and extraction of pectinases obtained by solid state fermentation of agroindustrial residues with *Aspergillus niger* . Biores Technol 71: 45–50.

[3] Cunha AG, Fernandez-Lorente G, Gutarra MLE, Bevilaqua JV, Almeida RV, et al (2009) Separation and immobilization of lipase from *Penicillium simplicissimum* by selective adsorption on hydrophobic supports. Appl Biochem Biotechnol 156: 563–575. 10.1007/s12010-008-8425-7 19037600

[4] Díaz AB, Caro I, Ory I, Blandino A (2007) Evaluation of the conditions for the extraction of hydrolitic enzymes obtained by solid state fermentation from grape pomace. Enzyme Microb Technol 41: 302–306.

[5] Gekko K, Timasheff SN (1981) Mechanism of Protein Stabilization by Glycerol: Preferential Hydration in Glycerol-Water Mixtures. Biochemistry 20: 4667–4676. 7295639 10.1021/bi00519a023

[6] Godoy MG, Gutarra MLE, Castro AM, Machado OLT, Freire DMG (2011) Adding value to a toxic residue from the biodiesel industry: production of two distinct pool of lipases from *Penicillium simplicissimum* in castor bean waste. J Ind Microbiol Biotechnol 38: 945–953. 20844923 10.1007/s10295-010-0865-8

[7] Godoy MG, Gutarra MLE, Maciel FM, Felix SP, Bevilaqua JV, et al (2009) Low cost methodology for biodetoxification of castor bean waste and lipase production. Enzyme Microb Technol 44: 317–322.

[8] Gutarra MLE, Cavalcanti EDC, Freire DMG, Castilho LR, Sant'anna GL Jr (2005) Lipase production by solid state fermentation: cultivation conditions and operation of a packed-bed bioreactor. Appl Biochem Biotech 121: 105–116. 10.1385/abab:121:1-3:0105 15917592

[9] Gutarra MLE, Godoy MG, Maugeri F, Rodrigues MI, Freire DMG, et al (2009) Production of an acidic and thermostable lipase of the mesophilic fungus *Penicillium simplicissimum* by solid-state fermentation. Bioresour Technol 100: 5249–5254. 19560339 10.1016/j.biortech.2008.08.050

[10] Gutarra MLE, Godoy MG, Silva JN, Guedes IA, Lins U, et al (2009) Lipase production and *Penicillium simplicissimum* morphology in solid-state and submerged fermentations. Biotechnol Journal 4: 1450–1459. 10.1002/biot.200800298 19606429

[11] Heck JX, Hertz PF, Ayub MAZ (2005) Extraction optimization of xylanases obtained by solid-state cultivation of Bacillus circulans BL53. Process Biochem 40: 2891–2895.

[12] Jaeger KE, Dijkstra BW, Reetz MT (1999) Bacterial biocatalysts: molecular biology, three-dimensional structures and biotechnological applications of lipases. Annu Rev Microbiol 53: 315–351. 10547694 10.1146/annurev.micro.53.1.315

[13] Johri S, Verma V, Parshad R, Koul S, Taneja SC, et al (2001) Purification and characterisation of an ester hydrolase from a strain of *Arthrobacter* species: its application in asymmetrisation of 2-benzyl-1,3-propanediol acylates. Bioorg Med Chem 9: 269–273. 11249119 10.1016/s0968-0896(00)00240-6

[14] Laemmli UK (1970) Cleavage of structural proteins during assembly of head of bacteriophage-T4. Nature 227: 680–685. 5432063 10.1038/227680a0

[15] Li N, Zong M (2010) Lipases from the genus *Penicillium*: Production, purification, characterization and applications. J Mol Catal 66: 43–54.

[16] Mahadik ND, Puntambekar US, Bastawde KB, Khire JM, Gokhale DV (2002) Production of acidic lipase by *Aspergillus niger* in solid state fermentation. Process Biochem 38: 715–721.

[17] Mateo C, Palomo JM, Fernandez-Lorente G, Guisan JM, Fernandez-Lafuente R (2007) Improvement of enzyme activity, stability and selectivity via immobilization techniques. Enzyme Microb Technol 40: 1451–1463.

[18] Mitchell DA, Berovic M, Krieger N (2002) Overview of solid state bioprocessing. Biotechnol Annu Rev 8: 183–225. 12436920 10.1016/s1387-2656(02)08009-2

[19] Oliveira D, Di Luccio M, Faccio C, Rosa CD, Bender JP, et al (2004) Optimization of enzymatic production of biodiesel from castor oil in organic solvent medium. Appl Biochem Biotech 115: 771–780. 10.1385/abab:115:1-3:0771 15054231

[20] Palomo JM, Peñas MM, Fernández-Lorente G, Mateo C, Pisabarro AG, et al (2003) Solid-Phase Handling of Hydrophobins: Immobilized Hydrophobins as a New Tool To Study Lipases. Biomacromolecules 4: 204–210. 12625713 10.1021/bm020071l

[21] Salum TFC, Villeneuve P, Barea B, Yamamoto CI, Côcco LC, et al (2010) Synthesis of biodiesel in column fixed-bed bioreactor using the fermented solid produced by *Burkholderia cepacia*LTEB11. Process Biochem 45: 1348–1354.

[22] Sharma R, Chisti Y, Banerjee UC (2001) Production, purification, characterization, and applications of lipases. Biotechol Adv 19: 627–662. 10.1016/s0734-9750(01)00086-6 14550014

[23] Valente AM, Alexandre VM, Cammarota MC, Freire DMG (2010) Enzymatic hydrolysis of fat from fish industry effluents aimed at increasing methane production. Ciênc Tecnol Aliment 30: 483–488.

[24] Vardanega R, Remonatto D, Arbter F, Polloni A, Rigo E, et al (2010) Systematic study on extraction of lipase obtained by solid state fermentation of soybean meal by a newly isolated strain of *Penicillium sp* . Food Bioprocess Technol 3: 461–465..

[25] Viniegra-González G, Favela-Torres E, Aguilar CN (2003) Advantages of fungal enzyme production in solid state over liquid fermentation systems. Biochem Eng J 13: 157–167.

[26] Zeng GM, Shi JG, Yuan XZ, Liu J, Zhang ZB (2006) Effects of Tween 80 and rhamnolipid on the extracellular enzymes of *Penicillium simplicissimum* isolated from compost. Enzym Microb Tech 39: 1451–1456.

